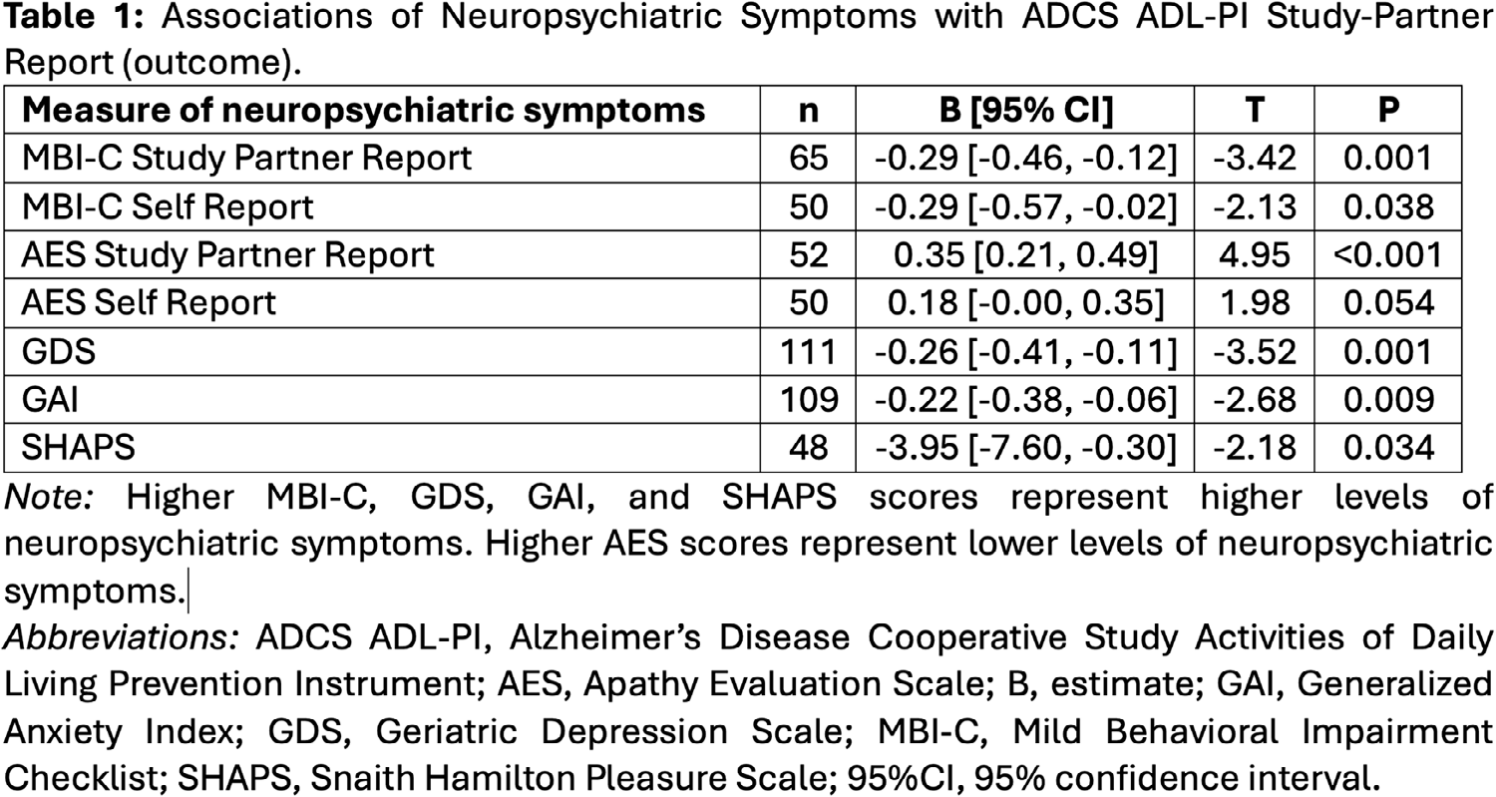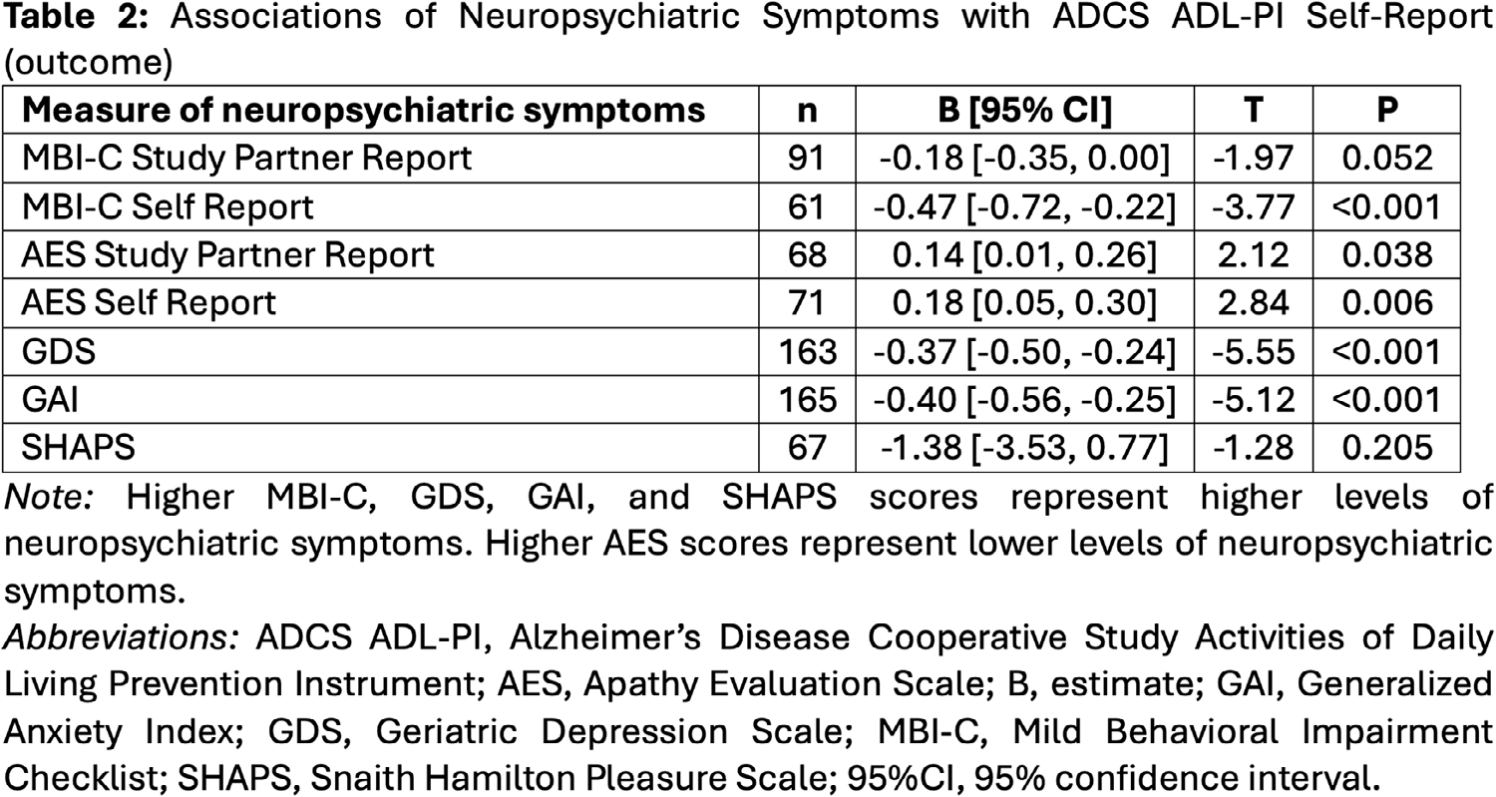# Impact of Neuropsychiatric Symptoms on Measures of Daily Functioning in Older Adults

**DOI:** 10.1002/alz70857_104488

**Published:** 2025-12-24

**Authors:** Mark A. Dubbelman, Grace Ma, Rebecca E. Amariglio, Catherine E Munro, Onyinye Udeogu, Madeline Yu, Claudia R. Aibel, Gad A. Marshall, Jennifer R. Gatchel

**Affiliations:** ^1^ Harvard Medical School, Boston, MA, USA; ^2^ Brigham and Women's Hospital, Boston, MA, USA; ^3^ Massachusetts General Hospital, Harvard Medical School, Boston, MA, USA; ^4^ Massachusetts General Hospital, Boston, MA, USA; ^5^ Mass General Brigham, Boston, MA, USA

## Abstract

**Background:**

Neuropsychiatric symptoms (NPS) in late‐life (e.g., changes in mood, motivation, sleep, and perception) may precede changes in cognition and indicate an underlying neurodegenerative process in the absence of noticeable cognitive decline. Changes in an older adult's ability to perform instrumental activities of daily living (IADLs; e.g., managing medications/finances, shopping and preparing meals) are often the first observable sign of cognitive impairment and may be affected by NPS. In this study, we examine associations between NPS and daily functioning reported by cognitively unimpaired older adults and their study‐partners, focusing on both overall symptoms and affective symptoms (e.g., depression, anxiety) typically seen in preclinical and prodromal stages. We hypothesize that increased self‐ and study‐partner‐reported NPS will be associated with worse daily functioning.

**Method:**

The sample included 177 community‐dwelling older adults without cognitive impairment or history of major psychiatric disorders (mean age 70.5±6.1 years; 63% female). All participants completed assessments of NPS (Mild Behavioral Impairment Checklist (MBI‐C), Apathy Evaluation Scale (AES), Geriatric Depression Scale (GDS), Geriatric Anxiety Index (GAI), and Snaith Hamilton Pleasure Scale (SHAPS)) and a measure of daily functioning (Alzheimer's Disease Cooperative Study ADL‐Prevention Instrument (ADCS ADL‐PI)). Study‐partners also completed the MBI‐C, AES, and ADCS ADL‐PI. We examined cross‐sectional relationships between participant and study‐partner‐reported functional and NPS assessments using separate linear regression models adjusted for age, sex, and education (in years).

**Result:**

Self‐reported MBI‐C, GDS, GAI, and self‐ and study‐partner‐reported AES, were found to have significant relationships with self‐reported ADCS ADL‐PI (Table 1). Additionally, study‐partner‐reported ADCS ADL‐PI scores were associated with the SHAPS, MBI‐C (self‐ and study‐partner‐reported), AES (study‐partner‐reported only), GDS, and GAI, such that worse IADL performance was correlated with greater NPS (Table 2).

**Conclusion:**

In cognitively unimpaired older adults, greater overall NPS and greater affective symptoms, including depression, anxiety, anhedonia, and apathy, were associated with worse participant and study‐partner‐reported daily functioning performance. This relationship underscores the importance of recognizing and treating NPS for improving daily functioning and quality of life, even prior to the onset of overt cognitive impairment, and considering source of report and methods of IADL measurement when utilizing the latter as clinical trial outcome measures.